# The Clinical and Diagnostic Spectrum of Esophagitis Dissecans Superficialis: A Comprehensive Retrospective Case Series and Literature Review

**DOI:** 10.7759/cureus.117820

**Published:** 2026-10-12

**Authors:** Btissam Essâdi, Ayoub Bouziane, Ouiam Elmqaddam, Hajar Koulali, Abdelkrim Zazour, Zahi Ismaili, Ghizlane Kharrasse

**Affiliations:** 1 Department of Hepato-gastroenterology, Mohammed VI University Hospital Center, Oujda, MAR; 2 Research Laboratory of Pathology and Digestive Oncology (LAPOD), Faculty of Medicine and Pharmacy, Mohammed First University, Oujda, MAR

**Keywords:** dysphagia, endoscopy, esophageal stricture, esophagitis dissecans superficialis, mucosal sloughing

## Abstract

Background and aim: Esophagitis dissecans superficialis (EDS), also known as sloughing esophagitis, is a rare and likely underdiagnosed desquamative disorder characterized by the exfoliation of large fragments of the esophageal squamous epithelium. Despite its dramatic endoscopic appearance, it is frequently misidentified as candidal or corrosive esophagitis. This study aims to provide a comprehensive analysis of the clinical spectrum, diagnostic hallmarks, and therapeutic outcomes of EDS through a Moroccan retrospective case series and a literature review.

Methods: We conducted a retrospective observational study of six patients diagnosed with EDS at a tertiary referral center between 2014 and 2025. Clinical, endoscopic, and histopathological data were analyzed alongside a narrative review of the literature conducted through PubMed up to December 2025, focusing on predisposing factors, diagnostic features, therapeutic approaches, and long-term outcomes.

Results: The cohort comprised four men and two women (mean age 59.3 ± 27.7 years). Dysphagia was a universal presenting symptom (100%), often accompanied by weight loss and vomiting. Endoscopic evaluation consistently revealed pathognomonic lamellar or sheet-like mucosal detachment and a "pseudo-tracheal" appearance, predominantly localized in the upper and middle esophageal thirds. Histology confirmed superficial desquamation and intraepithelial cleavage without evidence of malignancy. Potential predisposing factors varied across patients, with chronic medication exposure documented in four cases. Management involved high-dose proton pump inhibitors (PPIs) and withdrawal of offending agents. While 50% of patients achieved complete mucosal healing, the remainder experienced recurrences within one year, requiring iterative endoscopic dilations for symptomatic strictures.

Conclusion: EDS is a visually striking but fundamentally benign condition. Clinicians must maintain a high index of suspicion in elderly patients with polypharmacy or those with autoimmune triggers. Recognition of its characteristic endoscopic "cellophane tape" appearance and histopathological cleavage is essential to avoid aggressive interventions. When managed with trigger avoidance and mucosal protection, the overall prognosis is generally favorable.

## Introduction

Esophagitis dissecans superficialis (EDS), also referred to in the clinical literature as sloughing esophagitis, is an exceptionally rare and likely underdiagnosed desquamative disorder of the esophagus [1-3]. It is characterized by the sloughing of large fragments of the esophageal squamous mucosal lining, which may be expectorated or vomited as intact tubular or sheet-like casts [4-6]. First described by Birsch-Hirschfeld in 1877 and subsequently named by Rosenberg in 1892, EDS remained a diagnostic curiosity for more than a century [7-10]. With the widespread adoption of upper gastrointestinal endoscopy, increasing numbers of cases have been identified; nevertheless, the reported incidence remains exceedingly low, ranging from 0.03% to 0.06% in large retrospective series [3,4,9-11].

The clinical significance of EDS lies in its capacity to mimic far more ominous esophageal pathologies, including corrosive injury and chemical burns, frequently prompting unnecessary and aggressive diagnostic or therapeutic interventions. Despite its dramatic endoscopic appearance, the terminology surrounding this entity has historically been inconsistent, with descriptors such as esophagitis exfoliativa and epithelial necrolysis used interchangeably. Current consensus favors the term sloughing esophagitis when the underlying mechanism is predominantly irritant-related, whereas EDS remains the preferred designation in idiopathic or autoimmune-mediated cases [12,13].

Although its endoscopic presentation is often striking and alarming, EDS is generally regarded as a benign, self-limiting condition that resolves without permanent pathological sequelae [1-3,14,15]. However, its clinical manifestations are highly heterogeneous, ranging from asymptomatic incidental findings to debilitating dysphagia, odynophagia, and significant upper gastrointestinal hemorrhage [6,7,10,11,16,17]. The precise pathogenesis remains incompletely understood, yet strong associations have been documented with mucosal irritants, pharmacological agents, mechanical trauma, and autoimmune bullous dermatoses [1,3,6,11,16,18,19].

The present work combines original clinical data from a Moroccan cohort with a comprehensive analysis of published cases, aiming to provide a robust and integrative scientific overview of this enigmatic entity.

Study objectives

The primary objective of this study was to characterize the clinical, endoscopic, and histopathological features of EDS in a Moroccan case series. Secondary objectives were to describe therapeutic management and longitudinal outcomes, including recurrence and esophageal stricture formation, and to contextualize our findings through a narrative review of the available literature.

## Materials and methods

Patient selection and study framework

We conducted a retrospective case series of all documented cases of EDS identified at our tertiary referral center. Our review spanned more than a decade, from December 2014 through May 2025, during which our team performed a total of 9,991 upper gastrointestinal endoscopies. To ensure the robustness of our data, we systematically screened this entire procedural volume, including all patients who met the diagnostic criteria for EDS within the study period.

Clinical and endoscopic data synthesis

Once we identified the cohort, we conducted a granular review of standardized medical records. Our data extraction went beyond basic demographics; we focused on the nuances of clinical presentation, identifying potential triggers and underlying comorbidities that might influence disease progression.

The endoscopic review was particularly rigorous. Rather than relying on high-level summaries, we scrutinized the specific distribution and morphological nuances of mucosal sloughing in each case. To safeguard against diagnostic error, we cross-referenced these findings with histopathological reports, specifically aiming to differentiate EDS from its frequent clinical mimics, such as infectious or neoplastic esophagitis.

Diagnostic criteria

The diagnosis of EDS was based on the presence of characteristic endoscopic findings, particularly lamellar or sheet-like sloughing of the superficial esophageal mucosa, in conjunction with compatible histopathological findings, including superficial squamous desquamation and/or intraepithelial cleavage. Histopathological assessment was also used to exclude important differential diagnoses, particularly infectious and neoplastic esophagitis.

Therapeutic monitoring and long-term outcomes

Our analysis also tracked the diverse management paths taken by clinicians, from the cessation of suspected offending medications to the use of immunosuppressants and targeted endoscopic therapy. Because EDS can be a chronic or recurrent condition, we prioritized longitudinal follow-up. This allowed us to map the clinical trajectory of the disease, recording not only the resolution of symptoms but also the incidence of recurrence and the development of structural complications, such as esophageal strictures or hemorrhage.

Literature review strategy

A narrative literature review was conducted using PubMed, including publications available up to December 2025. The review focused on publications addressing EDS and its clinical presentation, endoscopic findings, histopathological characteristics, potential etiologies, therapeutic management, and outcomes. Relevant case reports, case series, and review articles providing clinical or diagnostic information applicable to the objectives of the present study were considered. Duplicate publications and reports without sufficient clinical or diagnostic information were not retained. Reference lists of relevant publications were also reviewed to identify additional pertinent reports.

Ethical oversight

This retrospective study was based on the analysis of anonymized, existing medical records. No direct patient contact occurred, and no identifying patient information was accessed or included in the manuscript.

## Results

Patient characteristics

During the study period, six patients with EDS were identified. The cohort included four men (66.7%) and two women (33.3%), with a mean age of 59.3 ± 27.7 years (range: 22-95 years). Two patients had a history of diabetes mellitus, one patient had arterial hypertension with a prior parotid adenocarcinoma, and one patient had a neuropsychiatric history. Two patients had no relevant medical history. Chronic medication use was documented in four patients, including insulin, calcium channel blockers, and anxiolytic agents.

Clinical presentation

Dysphagia was the presenting complaint in all patients (100%). Associated symptoms were frequently reported and included weight loss in four patients, vomiting in one patient, and regurgitation with hypersialorrhea in another. The duration of symptoms prior to diagnosis was highly variable, ranging from two months to three years. The median symptom duration was 10 months, reflecting the often insidious and delayed clinical recognition of the disease.

Endoscopic findings

Upper gastrointestinal endoscopy revealed characteristic features of EDS in all cases. The most common endoscopic pattern consisted of annular esophageal narrowing (Figure 1) associated with lamellar or sheet-like mucosal detachment (Figure 2). In several patients, the detached mucosa appeared as large, whitish fragments loosely adherent to the underlying esophageal wall. A pseudo-tracheal or sclerous aspect of the mucosa was observed in some cases.

**Figure 1:**
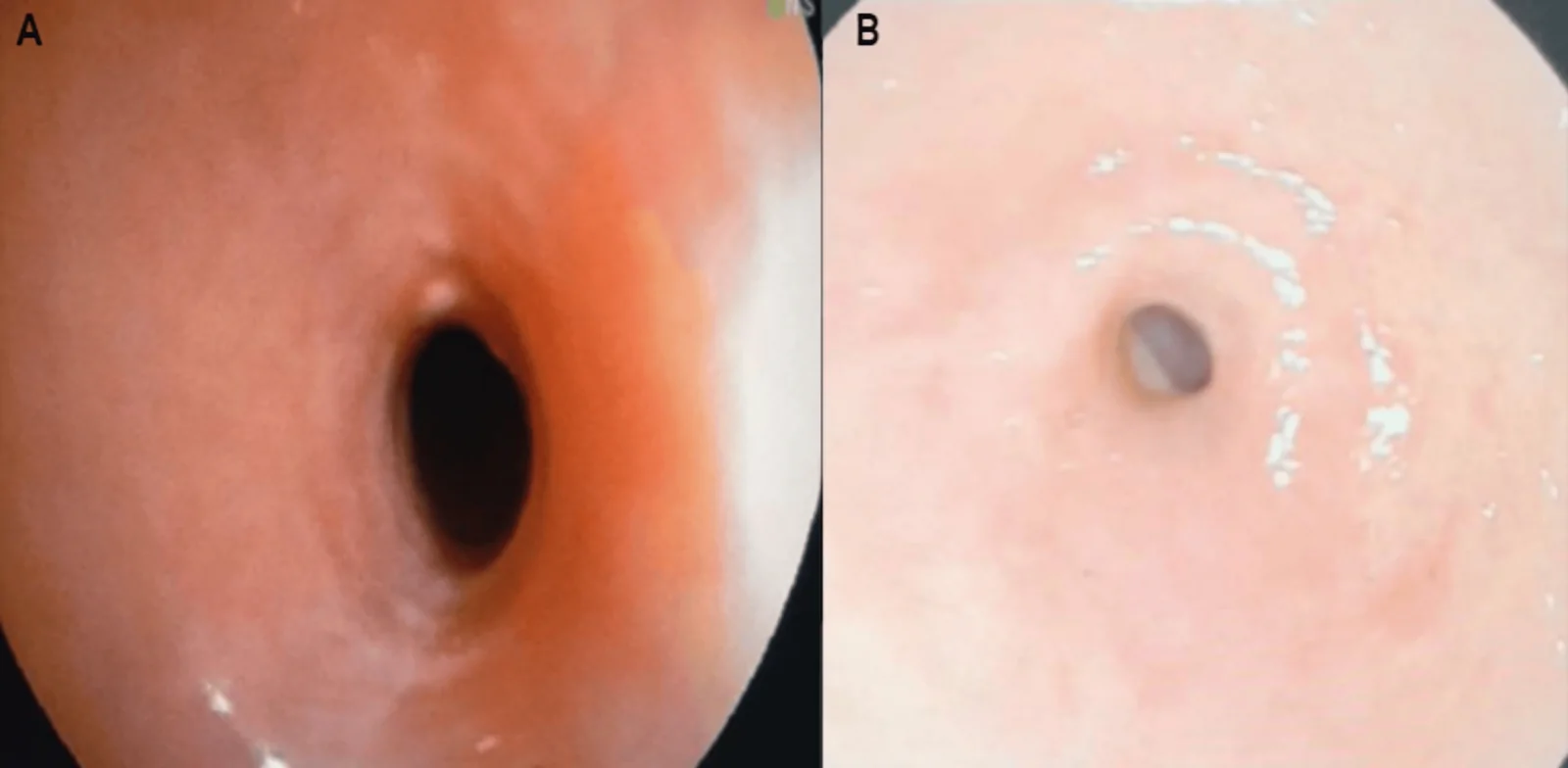
Endoscopic appearance of acquired esophageal stenosis in esophagitis dissecans superficialis. (A) Annular narrowing of the esophageal lumen. (B) Additional endoscopic view showing marked reduction in luminal caliber.

**Figure 2:**
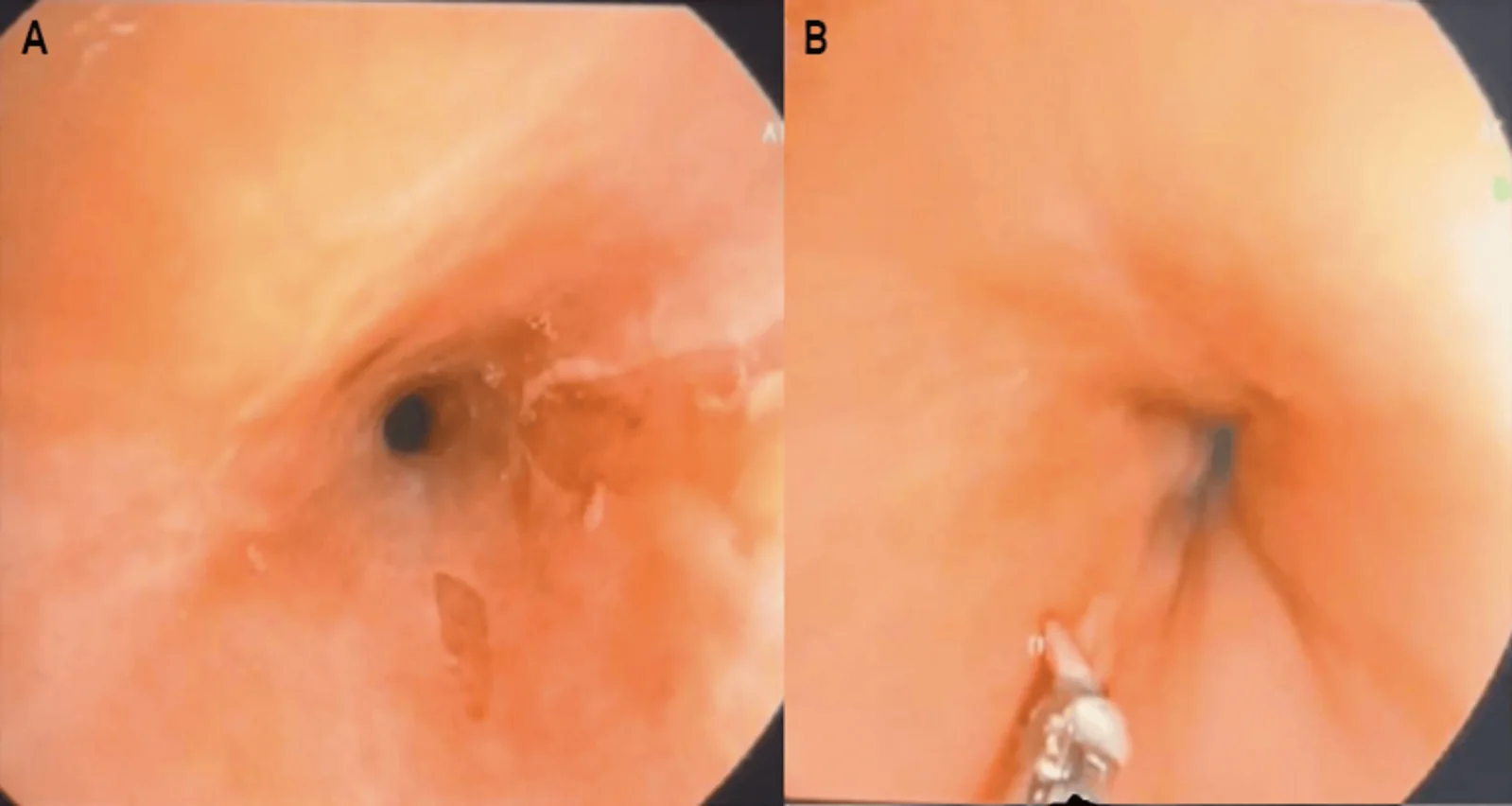
Endoscopic features of mucosal sloughing in esophagitis dissecans superficialis. (A) Lamellar detachment of the superficial esophageal mucosa. (B) Sheet-like mucosal sloughing with a detached mucosal flap.

Lesions were predominantly located in the upper (66.66%) and middle thirds (33.33%) of the esophagus, with no isolated distal involvement observed. Despite the extensive mucosal sloughing, none of the patients exhibited active endoscopic bleeding at the time of diagnosis.

Histopathological findings

Endoscopic biopsies were performed in all patients. Histological examination consistently demonstrated superficial desquamation of the squamous epithelium and/or intraepithelial cleavage. Representative histological images show detached fragments of superficial esophageal squamous mucosa with intraepithelial splitting and focal superficial parakeratosis (Figure 3). An additional representative section demonstrates the characteristic two-toned appearance, with a detached superficial pale eosinophilic/parakeratotic sloughed layer overlying more basophilic viable squamous epithelium, separated by intraepithelial cleavage (Figure 4). These changes were accompanied by a chronic, nonspecific inflammatory infiltrate. No fibrinopurulent exudates were identified in any of the histopathological specimens. Importantly, no evidence of epithelial dysplasia or malignancy was identified in any specimen.

**Figure 3:**
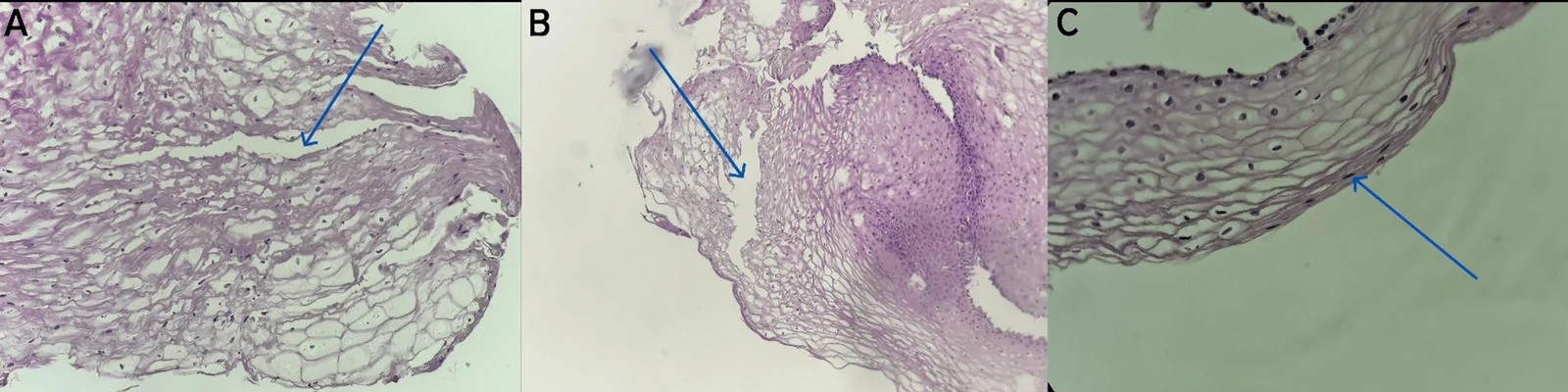
Histopathological features of esophagitis dissecans superficialis. (A) Detached fragments of superficial esophageal squamous mucosa showing intraepithelial splitting (blue arrow) (Hematoxylin-Eosin-Saffron (HES), ×10). (B) Intraepithelial cleavage with separation of the superficial squamous epithelial layers (blue arrow) (HES, ×20). (C) Higher-power view showing focal superficial parakeratosis (blue arrow) (HES, ×40).

**Figure 4:**
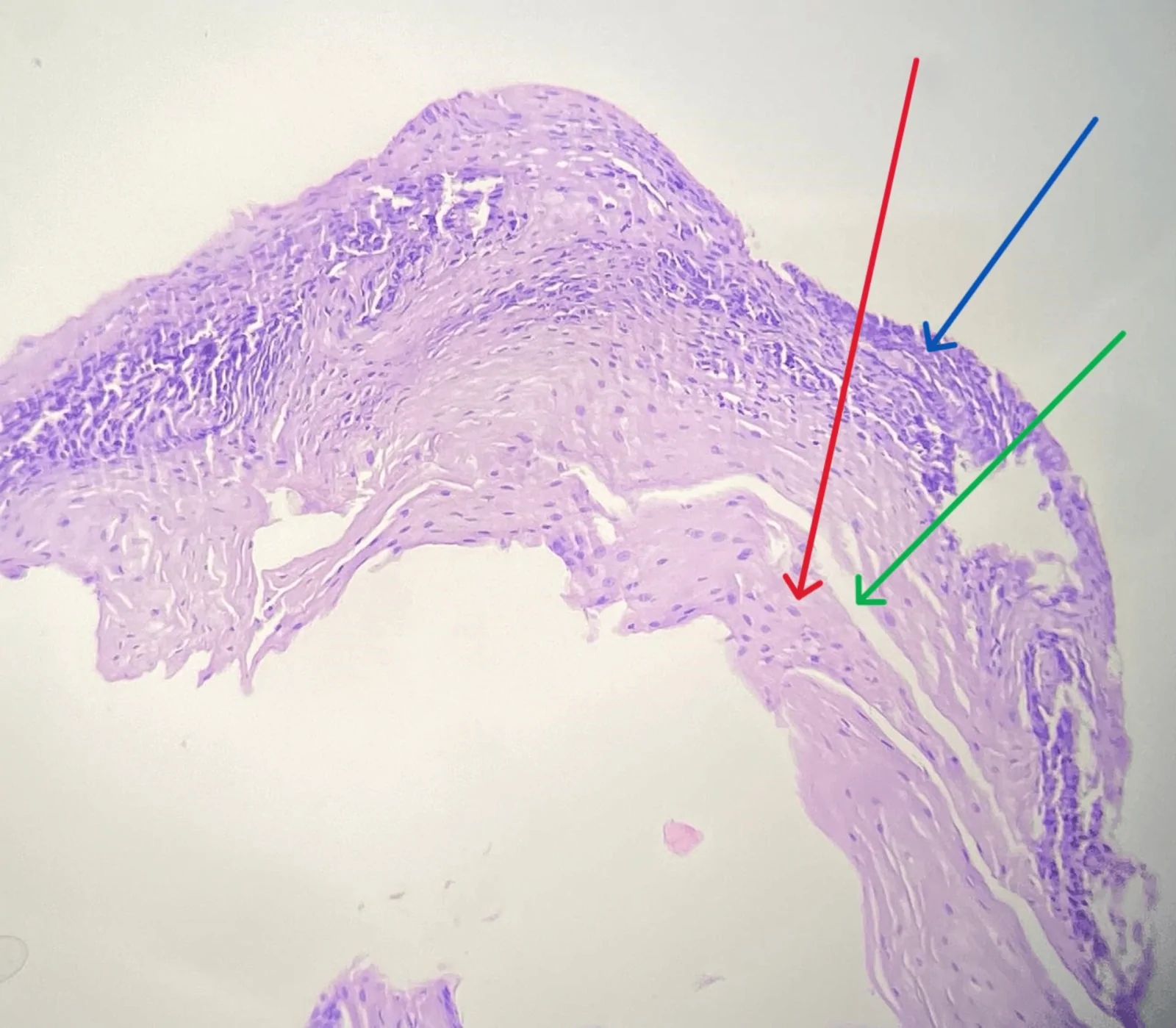
Histopathological examination showing the characteristic two-toned appearance of esophagitis dissecans superficialis. The red arrow indicates the detached superficial pale eosinophilic/parakeratotic sloughed layer; the blue arrow indicates the underlying more basophilic viable squamous epithelium; and the green arrow indicates the intraepithelial cleavage (split) separating the two layers (H&E, ×20).

Therapeutic management

All patients were managed conservatively. High-dose proton pump inhibitor (PPI) therapy (double the standard dose) was administered to all patients for a duration ranging from two to four months. Additional supportive measures included dietary modification and withdrawal of potential mucosal irritants or suspected offending medications when applicable.

Two patients (33.3%) developed clinically significant annular esophageal narrowing requiring endoscopic dilation. One patient underwent three dilation sessions, while the second required seven sessions due to persistent dysphagia. No procedural complications were reported.

Clinical and endoscopic outcomes

During follow-up, three patients (50%) achieved complete clinical and endoscopic remission, with full restoration of normal esophageal mucosa. The remaining three patients (50%) experienced disease recurrence, with recurrence occurring between six months and one year after the initial diagnosis. Repeat endoscopic biopsies were obtained in patients with recurrence. Histopathological examination showed no significant changes compared with the initial biopsies, with persistence of the characteristic features of EDS and no evidence of dysplasia or malignant transformation. In these patients, recurrent dysphagia was successfully managed with repeated endoscopic dilations and continuation of PPI therapy.

No severe complications-including perforation, significant hemorrhage, or malignant transformation-were observed during the follow-up period. Overall, disease control was achieved in all patients, confirming the favorable prognosis of EDS when appropriately recognized and managed. The clinicopathological characteristics and longitudinal outcomes of the cohort are summarized in Table 1.

**Table 1:** Clinicopathological Characteristics and Longitudinal Outcomes of Our Cohort (n = 6) PPI: proton pump inhibitor

Domain	Variable	Finding
Demographics	Number of patients, n	6
	Age, mean ± SD (years)	59.3 ± 27.7
	Age range (years)	22-95
	Male sex, n (%)	4 (66.7%)
	Female sex, n (%)	2 (33.3%)
Clinical presentation	Dysphagia, n (%)	6 (100%)
	Weight loss, n	4
	Vomiting, n	1
	Regurgitation/hypersialorrhea, n	1
	Median symptom duration (months)	10
	Symptom duration range (months)	2-36
Endoscopic findings	Mucosal appearance	Lamellar or sheet-like mucosal sloughing
	Annular esophageal narrowing	Observed
	Pseudo-tracheal/sclerous appearance	Observed
	Upper-third involvement, n (%)	4 (66.7%)
	Middle-third involvement, n (%)	2 (33.3%)
	Active bleeding at diagnosis	None
Histopathology	Superficial squamous desquamation	Present
	Intraepithelial cleavage	Present
	Inflammatory pattern	Chronic, nonspecific
	Dysplasia or malignancy	None detected
Management	High-dose PPI therapy, n (%)	6 (100%)
	Duration of PPI therapy (months)	2-4
	Endoscopic dilation, n (%)	2 (33.3%)
	Three dilation sessions, n	1
	Seven dilation sessions, n	1
Outcomes	Complete mucosal healing, n (%)	3 (50%)
	Recurrence, n (%)	3 (50%)
	Time to recurrence (months)	6-12
	Severe complications	None

## Discussion

In our cohort, the mean age was 59.3 years, with a clear male predominance (66.7%). This male predominance contrasts with several Western series reporting a slight female predominance, particularly in cases linked to psychoactive medications [12,13]. The observed age distribution confirms that although EDS primarily affects older individuals-likely due to polypharmacy and cumulative mucosal vulnerability-it may also occur in younger adults following acute chemical exposure or severe mechanical trauma.

The etiopathogenesis of EDS is multifactorial and generally reflects direct compromise of squamous epithelial integrity. A substantial proportion of patients are exposed to more than five medications, implicating chronic debilitation and pill-induced mucosal injury [11,13]. Agents such as alendronate, aspirin, and diclofenac are well-established contributors [1,3-6,16,19,20].

Direct oral anticoagulants have recently emerged as implicated agents. Dabigatran, containing a tartaric acid core, exerts direct chemical injury [3,21], while apixaban, although lacking tartaric acid, includes sodium lauryl sulfate, a surfactant known to induce mucosal irritation and desquamation [9]. Selective serotonin reuptake inhibitors (SSRIs) and serotonin-norepinephrine reuptake inhibitors (SNRIs) (e.g., fluoxetine, sertraline) have also been associated with EDS in 50%-73% of cases in selected series, possibly through direct epithelial effects [11-13]. Ferrous sulfate deposition may also cause focal mucosal erosion and sloughing, supported by iron-positive staining in the basal epithelial layer [22].

Paraquat, on the other hand, is profoundly corrosive to human tissue. Through the generation of reactive oxygen species, it induces lipid peroxidation, overwhelming oxidative stress, and rapid mucosal necrosis [7]. EDS in this context is often tubular, reflecting near-total exfoliation of the esophageal lining [7]. Ingestion of excessively hot liquids, explosive thermal exposure, or mechanical trauma can precipitate EDS [23].

The strongest systemic association of EDS is with autoimmune bullous dermatoses, particularly pemphigus vulgaris and bullous pemphigoid [1,24]. In pemphigus vulgaris, autoantibodies target desmoglein-1 and desmoglein-3, essential components of intercellular adhesion [8,24]. In mucosal epithelium, where desmoglein-1 expression is limited, desmoglein-3 antibodies alone are sufficient to induce suprabasal acantholysis [8]. Esophageal involvement occurs in 46%-87% of pemphigus vulgaris patients and may manifest as an esophageal Nikolsky sign-detachment of normal-appearing mucosa upon minimal mechanical contact [8]. Rare cases of bullous pemphigoid-associated EDS have been reported following SARS-CoV-2 vaccination, likely mediated by molecular mimicry [2].

In line with the clinical patterns described in the literature, dysphagia was a constant symptom in our patients. The frequent association with weight loss, vomiting, or regurgitation observed in our cohort further mirrors previously reported symptomatic clusters. Notably, the wide interval between symptom onset and diagnosis in our patients-ranging from two months to three years-highlights the insidious nature of the disease and echoes prior observations of delayed recognition, particularly in the absence of alarm signs or overt mucosal bleeding.

Endoscopically, EDS is characterized by "cellophane tape" mucosa-thin, translucent membranes partially detached from the esophageal wall [11,12]. Three reproducible patterns emerge: striping, cast formation, and a post-slough erosive phase. Importantly, the absence of significant bleeding despite extensive desquamation distinguishes EDS from viral or severe peptic esophagitis [11,12].

The endoscopic features identified in our cohort closely replicate these characteristics. Annular narrowing associated with lamellar mucosal detachment and a pseudo-tracheal, sclerous aspect constituted the predominant findings, with preferential involvement of the upper and middle thirds of the esophagus. Importantly, despite extensive mucosal sloughing, none of our patients exhibited significant endoscopic bleeding.

Furthermore, EDS frequently mimics candidal esophagitis [3,6,11,16,25,26]. While both present with white plaques, EDS membranes are diffuse, vertically oriented, and sheet-like, whereas Candida plaques are typically focal, granular, and easily scraped [3,25,26].

Hart et al. proposed three diagnostic endoscopic criteria [12]: sloughed mucosal strips exceeding 2 cm, normal-appearing underlying mucosa, and absence of ulceration or friability in adjacent tissue.

Adequate biopsy sampling remains essential, with some cases requiring up to 10 specimens to demonstrate intraepithelial clefting and parakeratosis, features that reliably differentiate EDS from eosinophilic and infectious esophagitides [12,13,25]. Direct and indirect immunofluorescence are indispensable when autoimmune etiologies are suspected [8,18,24].

Histopathological findings in our series were uniformly consistent with those described in the literature. Superficial epithelial desquamation and intraepithelial clefting were observed in all cases, accompanied by chronic, nonspecific inflammatory changes. No fibrinopurulent exudates were identified in our cohort. Furthermore, repeat biopsies obtained in patients with recurrence showed no significant histopathological changes compared with the initial findings, with persistence of the characteristic features of EDS. Crucially, no dysplasia or malignant transformation was identified in any specimen. The clinical presentation, suspected etiologies, histopathological findings, and treatment outcomes reported in the literature are summarized in Table 2.

**Table 2:** Clinical Presentation, Etiology, and Treatment Outcome NSAID: nonsteroidal anti-inflammatory drug, PPI: proton pump inhibitor, PBSCT: peripheral blood stem cell transplantation

Author and Year	Age/Gender	Primary Symptoms	Endoscopic Findings	Histopathological Characteristics	Suspected Etiology	Treatment and Outcome
Kanagalingam et al., 2021 [5]	70 / Male	Multiple episodes of coffee-ground emesis (GI hemorrhage).	White vertical strip of detached squamous mucosa, severe erosive esophagitis, radiation esophagitis.	Fibrinopurulent exudate and rare reactive squamous cells.	Multifactorial (metastatic hepatocellular carcinoma, radiation therapy, and tyrosine kinase inhibitor lenvatinib).	Acid-suppressive therapy (pantoprazole 40 mg twice daily) and sucralfate; no recurrence of bleeding.
Altaiam et al., 2025 [24]	45 / Male	Progressive oral ulceration, inability to tolerate intake, 10 kg weight loss, odynophagia/dysphagia.	White desquamation of esophageal mucosa, glove-like peeling, mucosal flap.	Stratified squamous epithelium with suprabasal acantholysis, “tombstone appearance” of the basal layer, mixed inflammatory infiltrate (eosinophils, lymphocytes).	Mucosal-dominant pemphigus vulgaris (PV).	Systemic oral prednisolone (tapering dose) and planned azathioprine; marked clinical improvement.
Iwamuro et al., 2020 [19]	18 / Female	Vomiting blood.	Extensive sloughing of esophageal mucosa and fresh blood.	Necrotic tissues and degenerative squamous cells.	Occurring after autologous PBSCT.	Fasting, IV lansoprazole, blood transfusion; healed on day 7.
Iwamuro et al., 2020 [19]	29 / Male	Melena and hematochezia.	Bloody lump in the middle/lower esophagus, mucosal sloughing, and hematoma.	Not reported in the source.	Following haploidentical PBSCT.	Fasting, IV omeprazole, sodium alginate, blood/platelet transfusion; healed on day 7.
Iwamuro et al., 2020 [19]	21 / Male	Hematemesis.	Fresh blood and peeled esophageal mucosa.	Not reported in the source.	Following haploidentical PBSCT.	Fasting, IV omeprazole, platelet transfusion; healed on day 5.
Iwamuro et al., 2020 [19]	16 / Male	Hematemesis.	Bloody mass in the esophagus, membranous peeled-off lower end.	Not reported in the source.	Following HLA-1-locus mismatched PBSCT.	Fasting, IV omeprazole, blood/platelet transfusion; persistent lesions on day 7.
Ng et al., 2021 [25]	59 / Male	Recurrent food bolus impaction (seven episodes over two years), odynophagia, dysphagia.	Short strictures, white patches resembling candidiasis, vertical lines, and ulceration.	Intraepithelial splitting, parakeratosis, superficial necrosis, neutrophil infiltration, and microabscesses.	Possible association with rheumatoid arthritis medications (hydroxychloroquine/sulfasalazine) or repeated mechanical trauma from balloon dilation.	High-dose esomeprazole (40 mg), pureed diet, and a tapering course of prednisolone (40 mg); no readmission for food bolus to date.
Senyondo et al., 2022 [15]	54 / Male	Sharp epigastric pain, nausea, intractable vomiting, 20-pound weight loss.	Esophageal mucosal sclerosis.	Splitting of squamous epithelium, thick layer of parakeratosis, mild basal cell hyperplasia, mild intraepithelial neutrophilic infiltrate.	Chronic use of oral diclofenac (NSAID).	Discontinuation of diclofenac, oral sucralfate, and high-dose pantoprazole; symptoms resolved after one week.
Baran et al., 2023 [17]	17 / Female	Chronic nausea (daily), vomiting (one to two times/week), constipation.	Sloughing of mucosa with loss of vascular pattern, vertical lines of detaching white mucosa.	Chronic esophagitis with partial superficial squamous mucosal necrosis and sloughing; two-toned appearance.	Suspected association with psychoactive medications (fluoxetine).	Not reported in the source; discussion notes that spontaneous resolution is typical.
Qasim et al., 2023 [11]	62 / Female	Severe epigastric pain, sharp nausea, vomiting, dysphagia to solids.	Columns of sloughed mucosa in the lower third of the esophagus.	Intraepithelial lymphocytes without eosinophils; inflammatory infiltrates and epithelial regeneration.	Possible association with underlying systemic lupus erythematosus (SLE).	Omeprazole 40 mg daily for eight weeks; significant clinical improvement.
Tao et al., 2021 [7]	6 Male / 9 Female	Sore throat or dysphagia (100%), nausea and vomiting (86.7%), heartburn or upper abdominal pain (73.3%), hematemesis (60%).	Not reported in the source; patients did not undergo endoscopy, and mucosal casts were vomited.	Extensive injury, necrosis, and hemorrhage of digestive tract epithelium; separation at the suprabasal level (tombstone-like basal cells).	Acute transoral paraquat poisoning.	Gastrointestinal lavage, high-dose steroids, blood perfusion; 12 deaths, three survivals.
Relvas et al., 2025 [1]	42 / Female	24-hour history of hematemesis, sensation of “tearing” of the esophagus, retrosternal pain.	Hyperemic or hemorrhagic areas, sloughing mucosa, adjacent whitish fibrinous membranes, submucosal blisters, erythematous distal esophagus.	No changes (biopsies performed two weeks after presentation).	Secondary to mechanical trauma (accidentally swallowing a piece of bone).	PPI; complete healing shown on control endoscopy after two weeks.
Coppola et al., 2000 [27]	78 / Female	Solid food dysphagia (since 1992), heartburn.	Erythema, friability, detachment of large fragments of squamous mucosa, strictures.	Detached squamous epithelium with edematous suprabasal clefts, chronic T-lymphocyte infiltration, decreased/abnormal desmosomes (electron microscopy).	Chronic esophagitis dissecans (CED) associated with strictures.	Lansoprazole 30 mg daily and gradual dilation; outcome not fully specified.
Jaben et al., 2019 [28]	71 / Male	Chest pain, regurgitation, mild odynophagia, coughing up mucosal tissue and blood.	Severe, circumferential esophagitis with deep, serpiginous ulcerations and mucosal sloughing.	Parakeratosis, minimal inflammation, basal cell hyperplasia.	Idiopathic.	High-dose corticosteroids (IV methylprednisolone followed by oral prednisone) and omeprazole; dysphagia and odynophagia resolved.
Zaher et al., 2023 [14]	81 / Female	Dizziness, generalized weakness, poor appetite, 8-pound weight loss in two weeks.	Severe mucosal changes characterized by sloughing in the lower third of the esophagus.	Parakeratosis, hyperkeratosis, and coagulative necrosis.	Idiopathic.	High-dose PPI therapy; symptom improvement and diet advancement.
Escobar Ortiz et al., 2025 [29]	62 / Female	Pyrosis (heartburn).	Extensive whitish sheets of detached superficial mucosa involving the whole circumference of the mid-esophagus.	Reactive squamous epithelium separated from basement membrane, focal polymorphonuclear infiltration.	Unknown (idiopathic).	Antisecretory treatment; follow-up endoscopy demonstrated mucosal healing.
Then et al., 2021 [3]	65 / Female	Nausea, vomiting, odynophagia, throat pain, dysphonia.	Mild superficial mucosal desquamation in the entire esophagus.	Not reported in the source.	Incidental hair dye ingestion containing resorcinol and para-phenylenediamine (PPD).	Intravenous pantoprazole, diet advanced to solids; symptoms resolved.
Orosz and Patel, 2021 [6]	29 / Male	Episodes of hematemesis, epigastric pain, gastroesophageal reflux.	Sloughing of mucosa into sheets and casts in the distal esophagus.	Not reported in the source.	Alcohol abuse and irritants.	Bowel rest and acid suppression; symptoms resolved over two days.

There is no standardized treatment algorithm for EDS; management is largely supportive [1,10,11,14]. Withdrawal of offending agents is paramount and often results in rapid clinical improvement [1,3,6,11,14,27]. Acid suppression with high-dose PPIs is widely employed to protect the exposed mucosa rather than treat the underlying desquamation [1,6,11,14]. Immunosuppression is essential in autoimmune-mediated cases [11,24,28]. Endoscopic dilation is required in a minority of patients who develop residual strictures [11,27].

From a therapeutic standpoint, all patients in our cohort were managed conservatively with high-dose PPI therapy. Two patients developed symptomatic annular strictures requiring endoscopic dilation, with repeated sessions necessary in one case. During follow-up, complete mucosal healing was achieved in half of the patients, while the remainder experienced recurrence within six to 12 months. Importantly, recurrent disease was successfully controlled with repeated dilations and continuation of acid suppression, and no severe complications were observed. These outcomes further confirm the generally favorable prognosis of EDS when appropriately recognized and managed.

Overall, the prognosis is excellent, with mucosal healing typically occurring within one to eight weeks [1,6,11,19,29]. In contrast, mortality in paraquat-associated cases reflects multisystem organ failure rather than esophageal injury itself [7].

A major strength of our study is the integrated assessment of clinical, endoscopic, histopathological, therapeutic, and longitudinal findings in a Moroccan cohort, providing additional data from a region where EDS remains scarcely reported. Longitudinal follow-up allowed us to document both recurrence and stricture-related outcomes, including the histopathological findings obtained during recurrent disease. In addition, the accompanying narrative review places our observations within the broader spectrum of previously reported clinical presentations, etiologies, histopathological features, and therapeutic approaches.

Limitations

This study has several limitations. First, its retrospective and single-center design, together with the small number of patients, limits the generalizability of our findings. The long study period may also have introduced variability in diagnostic assessment, histopathological sampling, therapeutic approaches, and follow-up. In addition, the retrospective nature of the medical records limited the uniform assessment of all potential predisposing factors and medication exposures. Finally, the available literature on EDS consists predominantly of case reports and small case series, which limits direct comparisons between studies and may introduce publication bias. Nevertheless, our series provides additional clinicopathological and longitudinal data on this rare and likely underdiagnosed condition, including follow-up histopathological findings in patients with recurrent disease.

## Conclusions

EDS is a rare and likely underdiagnosed disorder characterized by superficial esophageal mucosal sloughing. Recognition of its characteristic endoscopic and histopathological features is essential to distinguish it from infectious, corrosive, and neoplastic conditions. A careful review of potential triggers, including medications, chemical exposure, and autoimmune bullous diseases, should therefore form part of the diagnostic evaluation.

Management is primarily based on withdrawal of potential offending agents, acid suppression, and treatment of the underlying condition when identified. Although recurrence and esophageal strictures may occur, the overall prognosis is generally favorable, with symptomatic improvement and mucosal healing achievable with appropriate management.
